# Dietary Calcium and Risk for Prostate Cancer: A Case-Control Study Among US Veterans

**DOI:** 10.5888/pcd9.110125

**Published:** 2012-01-12

**Authors:** Christina D. Williams, Brian M. Whitley, Cathrine Hoyo, Delores J. Grant, Gary G. Schwartz, Joseph C. Presti, Jared D. Iraggi, Kathryn A. Newman, Leah Gerber, Loretta A. Taylor, Madeline G. McKeever, Stephen J. Freedland

**Affiliations:** Durham Veterans Affairs Medical Center. Dr Williams is also affiliated with Duke University Medical Center (DUMC), Durham, North Carolina; DUMC, DVAMC, Durham, North Carolina; DUMC, Durham, North Carolina; Julius L. Chambers-Biomedical/Biotechnology Research Institute, North Carolina Central University, Durham, North Carolina; Wake Forest University, Winston-Salem, North Carolina; Stanford University School of Medicine, Palo Alto, California; DUMC, DVAMC, Durham, North Carolina; DUMC, DVAMC, Durham, North Carolina; DUMC, DVAMC, Durham, North Carolina; DUMC, DVAMC, Durham, North Carolina; DUMC, DVAMC, Durham, North Carolina; DUMC, DVAMC, and Duke University School of Medicine, Durham, North Carolina

## Abstract

**Introduction:**

The objective of this study was to examine the association between calcium intake and prostate cancer risk. We hypothesized that calcium intake would be positively associated with lower risk for prostate cancer.

**Methods:**

We used data from a case-control study conducted among veterans between 2007 and 2010 at the Durham Veterans Affairs Medical Center. The study consisted of 108 biopsy-positive prostate cancer cases, 161 biopsy-negative controls, and 237 healthy controls. We also determined whether these associations differed for blacks and whites or for low-grade (Gleason score <7) and high-grade prostate cancer (Gleason score ≥7). We administered the Harvard food frequency questionnaire to assess diet and estimate calcium intake. We used logistic regression models to obtain odds ratios (ORs) and 95% confidence intervals (CIs).

**Results:**

Intake of calcium from food was inversely related to risk for prostate cancer among all races in a comparison of cases and biopsy-negative controls (*P *= .05) and cases and healthy controls (*P *= .02). Total calcium was associated with lower prostate cancer risk among black men but not among white men in analyses of healthy controls. The highest tertile of calcium from food was associated with lower risk for high-grade prostate cancer in a comparison of high-grade cases and biopsy-negative controls (OR, 0.37; 95% CI, 0.15-0.90) and high-grade cases and healthy controls (OR, 0.38; 95% CI, 0.17-0.86).

**Conclusion:**

Calcium from food is associated with lower risk for prostate cancer, particularly among black men, and lower risk for high-grade prostate cancer among all men.

## Introduction

In the Veterans Health Administration (VHA), there are approximately 12,000 incident cases of prostate cancer each year (LL Zullig, MPH, Durham VA Medical Center, unpublished data, March 2011). Environmental factors such as diet are thought to influence prostate cancer development and progression. Data on the effects of calcium intake on prostate cancer are inconsistent. Some epidemiologic studies provide evidence of a positive association (1-5), while others report no association (6-8). Nearly all of these studies were performed in populations made up predominantly of white men, even though associations between modifiable risk factors such as calcium intake and prostate cancer risk may differ by race.

A potential mechanism for the role of calcium in prostate cancer development and progression is that intracellular calcium controls the growth of prostate cancer cells and the process of apoptosis (9). Calcium may also have an indirect effect; it has been hypothesized that dietary calcium may increase prostate cancer risk by reducing circulating levels of 1,25-dihydroxyvitamin D (1,25[OH]_2_D) (10), which promotes the differentiation and inhibits the proliferation of prostate cells (11). Therefore, a high calcium intake would counteract the potentially anticarcinogenic effects of vitamin D and thereby promote tumor growth.

The objective of this study was to examine the relationship between calcium intake and prostate cancer risk and determine whether this association is different for blacks and whites or for low-grade and high-grade disease. We hypothesized that calcium would be positively associated with prostate cancer risk.

## Methods

### Study design

We used data from an ongoing case-control study of veterans screened for prostate cancer at the Durham Veterans Affairs Medical Center (DVAMC) in Durham, North Carolina. Details of this case-control study have been reported previously (12). This study was approved by the institutional review board at the DVAMC, and all patients provided written informed consent.

### Study participants

This study includes participants enrolled between January 2007 and September 2010 who were aged at least 18 years, had a prostate-specific antigen (PSA) screening test done within 12 months prior to enrollment, and had no prior history of prostate cancer. We identified men from the urology clinic at the DVAMC who were scheduled for a prostate biopsy because of an elevated PSA or abnormal rectal examination. Of the 785 men scheduled for a biopsy and screened for eligibility, 577 provided written consent to participate. Among participants who received the biopsy (n = 533), 216 were biopsy-positive and considered cases for this study; 316 were biopsy-negative and served as biopsy-negative controls. After we assessed eligibility by medical record review and obtained physicians' permission to contact patients, we recruited 393 healthy control participants (ie, no biopsy indication) from the urology and internal medicine clinics during routine visits. We required completion of study questionnaires for inclusion in the final analytic sample. Meeting this requirement were 50% of biopsy-positive cases, 51% of biopsy-negative controls, and 60% of healthy controls. Thus, the final sample consisted of 108 biopsy-positive cases, 161 biopsy-negative controls, and 237 healthy controls.

### Data collection

We collected diet and covariate data using self-administered questionnaires. We used the Harvard food frequency questionnaire (FFQ) for data on diet (13). Participants recalled their usual consumption of 61 foods and beverages in the previous 12 months. This FFQ has been tested for validity and found to be a good assessment of nutrient intake during a 1-year period (13). The FFQ also solicited information on dietary supplement use, including the frequency and dose of single supplements and multivitamins. Nutrient intakes were derived from the frequency, amount, and nutrient content of each food, beverage, and supplement on the FFQ. The Harvard School of Public Health conducted the nutrient analysis. We used a separate questionnaire to obtain information on potential prostate cancer risk factors, including smoking and alcohol use, physical activity, and family history of prostate cancer. To minimize differential recall bias due to biopsy results, we asked patients to complete questionnaires before the biopsy. The Gleason scores were based on standard reviews of biopsy specimens by a board-certified pathologist and were part of standard care. We abstracted Gleason scores and race information from the medical record. Trained personnel measured height and weight.

### Statistical analysis

We performed all analyses using SAS version 9.2 (SAS Institute, Inc, Cary, North Carolina). We examined total calcium intake (food plus supplements) and calcium from food only. We compared cases and controls by using a χ^2^ test for categorical variables and the Wilcoxon rank sum test for continuous variables. Calcium intake was adjusted for total calories using the nutrient residual method (14) and categorized into tertiles based on the distribution in the respective control population. Data from FFQs are useful for ranking nutrient intake; categorizing nutrient intake makes no assumption about the dose-response relationship between calcium and prostate cancer risk. We chose tertile categories because of the range of calcium intake in our study population. We examined tertiles separately for total calcium and tertiles for calcium from food only. We determined odds ratios (ORs) and corresponding 95% confidence intervals (CIs) through logistic regression to estimate relative risk for prostate cancer; we used the lowest tertile as the reference group. We modeled separately the risk for prostate cancer by using healthy controls and biopsy-negative controls. We examined the potential for effect modification by race in stratified analyses. We also entered a cross-product term in the models along with the main-effects terms to test for calcium-race interaction; we evaluated the coefficient of the cross-product term by using the Wald χ^2^ test. We used multinomial logistic regression to determine whether the association between calcium and prostate cancer varied by disease aggressiveness. These analyses compared the risk for low-grade prostate cancer (Gleason score <7, n = 60) relative to controls and the risk for high-grade (ie, aggressive) prostate cancer (Gleason score ≥7, n = 48) relative to controls. We adjusted all models for age (continuous), total calories (continuous), and race (white, black, other). Analyses with biopsy-negative controls were further adjusted for log-transformed PSA. We considered other potential confounders, including body mass index (BMI, kg/m^2^), family history of prostate cancer, smoking status, alcohol use, and vitamin D intake. These covariates did not appreciably alter our results and therefore were not included in the final models. We assessed linear trends in risk by incorporating into the models a continuous variable assigned the median nutrient intake for each tertile. *P* values less than .05 were considered statistically significant.

## Results

Cases and controls did not differ significantly by age, education, family history, smoking status, alcohol use, prevalence of supplement or vitamin use, or intakes of calcium or calories (Table 1). Compared with biopsy-negative controls, cases reported significantly less physical activity. Of cases, 56% of were black; of healthy controls, 35% were black. Healthy controls had a slightly higher mean BMI than cases (31 vs 29). The mean total calcium intake among our study participants was approximately 800 mg per day. Among biopsy-negative controls, the mean calcium intake (total and from food only) in blacks was significantly lower than in whites, and black healthy controls reported significantly less calcium from food than did white healthy controls (Table 2).

In a comparison of cases and biopsy-negative controls among all races, increasing calcium intakes from food but not total calcium was associated with lower risk for prostate cancer (*P* = .05) (Table 3). We found a significant interaction between race and total calcium (*P* = .04), which suggested that higher total calcium was linked with higher cancer risk in whites but lower risk in blacks, but we found no significant risk estimates in race-specific analyses (Table 3).

In a comparison of cases and healthy controls among all races, a larger intake of calcium from food but not total calcium was associated with lower risk for prostate cancer (Table 3). In race-specific analyses, total calcium was associated with lower prostate cancer risk among black men but not among white men. We found no statistically significant associations among whites. The interaction between total calcium and race was not significant (*P* = .07).

We found a moderate correlation between calcium and vitamin D (Spearman *ρ* = 0.59, *P* < .001 in healthy controls; Spearman *ρ* = 0.46, *P* < .001 in biopsy-negative controls); adjustment for vitamin D intake did not alter results.

We observed no associations between calcium intake (total or from foods only) and low-grade prostate cancer (Table 4). In a comparison of cases and biopsy-negative controls, the highest tertile of calcium from food was associated with lower risk for high-grade cancer. In a comparison of cases and healthy controls, the highest tertile of total calcium and of calcium from food was associated with lower risk for high-grade cancer.

## Discussion

We found little evidence to support a positive association between calcium intake and prostate cancer risk in this case-control study. On the contrary, we found no association between total calcium and prostate cancer risk and an inverse association between calcium from food and risk for prostate cancer among all men. An inverse association between total calcium and prostate cancer was limited to black men in analyses using healthy controls, although no evidence of an association was found among white men. Also, a high calcium intake correlated with lower risk for high-grade cancer but not low-grade cancer.

One meta-analysis reported that prospective cohort studies suggest a weak positive association between the highest and lowest category of calcium intake and prostate cancer risk and that case-control studies indicate no association (15). Theoretically, higher calcium intakes could increase prostate cancer risk by reducing the biologically active form of vitamin D, which can inhibit prostate cancer cell growth (16). This theory may explain, in part, the positive association between prostate cancer risk and high levels of calcium intake. Two prospective studies, for example, observed an elevated risk for prostate cancer for a calcium intake of 2,000 mg per day or more (1,17). The mean total calcium intake among our study participants was relatively low, approximately 800 mg per day. According to the US Department of Agriculture, an adequate calcium intake is 1,000 mg per day for men aged 51 to 70 years and 1,200 mg per day for men aged 70 or older (18). On the basis of these guidelines, only 27% of our study population had adequate calcium intake, so we did not have sufficient variation to test whether extremely high intakes (ie, ≥2,000 mg/d) correlated with prostate cancer risk. Our results suggest that among men with low to moderate calcium intake, an adequate calcium intake (ie, 1,000 mg/d) may reduce the risk for prostate cancer. Viewed alternatively, our study suggests that very low calcium intake may increase prostate cancer risk relative to adequate intake. Coupled with the data that high calcium intake may increase prostate cancer risk, our study supports the notion that most nutrients, particularly micronutrients and specifically calcium, may have a J-shaped or U-shaped relationship with disease, whereby deficiencies and excesses correlate with higher risk and adequate intakes correlate with lower risk (19).

In our study, calcium supplements contributed approximately 100 mg per day to total calcium in each participant group. Although total calcium intake may be a more informative measure than calcium intake from food only, we observed in analyses of all races inverse associations between prostate cancer and calcium from food but not total calcium. This finding suggests that calcium intake from supplements may not reduce prostate cancer risk as supplement users may expect and that adequate calcium from food sources alone may be sufficient to reduce prostate cancer risk. However, a level of supplemental calcium that could reduce prostate cancer risk and a level that could increase risk should be identified.

Few studies have examined whether associations between calcium and prostate cancer risk differ by race/ethnicity. Skin pigmentation has a strong effect on vitamin D status; people with darker skin have more melanin, which reduces the ability to synthesize vitamin D from sunlight radiation (20). As a result, blacks are more prone to vitamin D deficiency and reduced levels of calcium absorption (21). Our finding that blacks have lower calcium intake compared with whites is consistent with the literature (8,22). Our results further suggest that calcium intake affects prostate cancer risk differentially by race. The limited number of studies that have considered this possibility found no clear association between dietary calcium and prostate cancer risk among whites or blacks (8,23). One study, however, reported a correlation between an increase in dairy consumption and a higher risk for prostate cancer among whites but not blacks (23). In the same study, ORs for quartiles of calcium intake from food were less than 1 among blacks (*P* = .06) and greater than 1 among whites (*P* = .22), although ORs were not statistically significant (23). Our results also suggest an inverse association between calcium intake and prostate cancer risk among black men but not white men. These results may reflect the lower (but not significantly lower) caloric intake among blacks compared with whites, despite our attempt to control for total calories. Given that most studies of calcium and prostate cancer risk have included samples made up largely of white men (2,3,17,24) and that we show a difference in the effect of calcium on prostate cancer risk between black and white men, future studies are needed to validate our findings and understand the biological mechanisms responsible for our observations.

Dietary factors may impose different risks for subgroups of prostate cancers. Our results are consistent with the lack of an association between calcium and low-grade prostate cancer (8,25). In contrast to previous reports of null (8,24) and positive (25) associations with high-grade prostate cancer, we found an inverse association between high-grade prostate cancer and dietary calcium. Another study also noted lower risk for high-grade prostate cancer (defined as Gleason score 8-10) among men in the Prostate Cancer Prevention Trial who had a high calcium intake (26). Given the inverse association between calcium intake and prostate cancer risk we observed among black men, we considered the possibility that high-grade prostate cancer was more common in black case patients compared with white case patients and thus responsible for the inverse relationship between calcium intake and high-grade prostate cancer. However, in our study population, 44% of black men and 50% of white men with prostate cancer had high-grade prostate cancer. Again, our finding may imply that adequate calcium intake (ie, 1,000 mg/d) among people with a low- to moderate-calcium diet could reduce the risk for high-grade prostate cancer. We were unable to test the notion that a very high calcium intake may contribute to prostate cancer progression because our sample included few men who had a very high calcium intake.

This study had several limitations. The FFQ may not have included all foods necessary for accurately assessing intake, especially fortified foods and foods unique to certain geographic locations or racial/ethnic groups. This study had biases common to case-control studies. Nonresponse bias may have resulted from the large portion of participants who did not complete the study questionnaires and were excluded from analyses; thus, we cannot exclude the possibility that participants who completed the study questionnaires differed from those who did not. The FFQ required participants to recall their intake in the previous 12 months, which is likely not the etiologically relevant period of exposure, though the exact etiologically relevant time is not known. Recall bias could have been different for cases and controls. We attempted to minimize recall bias by interviewing men before their biopsy and biopsy results. Selection bias was minimized by recruiting all participants from a population of veterans screened for prostate cancer at the DVAMC, but bias is possible if some participants had previous biopsies or an elevated PSA or both. Our sample was small, resulting in limited statistical power and variation in nutrient intakes. Our study was based on data from veterans screened for prostate cancer and receiving care in the VA system, the largest health care system in the United States and an equal-access setting; therefore, generalizability of our findings to non-VA populations is uncertain. The major strength of our study is that the population of veterans at the DVAMC is particularly useful for examining racial disparities because of the equal-access health system and the large proportion of blacks receiving care at the DVAMC.

We observed lower risk for prostate cancer with increasing intakes of calcium from food in both healthy and biopsy-negative controls. The inverse association between total calcium and prostate cancer was limited to black men. Among all men, the highest calcium intake in our study was related to lower risk for high-grade prostate cancer but was not associated with low-grade prostate cancer. Overall, our findings suggest that among men with diets that have moderate to low calcium intake, adequate calcium intake may reduce the risk for prostate cancer, particularly among black men, and reduce the risk for high-grade prostate cancer among all men. Because of the numerous benefits of calcium in preventing chronic diseases, more research is needed to clarify its role in prostate health. In particular, researchers should determine the levels at which dietary calcium may increase the risk for prostate cancer and examine whether the effect of calcium on prostate cancer risk differs by race/ethnicity.

## Figures and Tables

**Table 1:** Participant Characteristics by Case-Control Status Among Veterans Screened for Prostate Cancer at Durham Veterans Affairs Medical Center, 2007-2010

Characteristic	Cases (n = 108)	Biopsy-Negative Controls (n = 161)	Healthy Controls (n = 237)	*P* Value^a^	*P* Value^b^
**Age, mean (SD), y**	63 (5.6)	63 (5.9)	62 (7.6)	.74	.12
**Race, no. (%)**					
Black	60 (56)	66 (41)	82 (35)	.15	.007
White	47 (43)	89 (55)	148 (62)		
Other	1 (1)	2 (1)	4 (2)		
Missing	0	4 (3)	3 (1)		
**≥College degree, no. (%)**	32 (30)	46 (29)	66 (28)	.90	.25
**BMI, mean (SD), kg/m^2^**	29 (5.3)	30 (5.2)	31 (5.2)	.38	.02
**Physical activity, mean (SD), MET h/wk**	12 (26)	21 (50.6)	10 (17)	.02	.70
**Family history of prostate cancer, no. (%)**	22 (20)	29 (18)	33 (14)	.63	.13
**Current smokers, no. (%)**	36 (33)	34 (21)	56 (24)	.06	.06
**Current drinkers, no. (%)**	54 (50)	64 (40)	98 (41)	.23	.30
**PSA, median, ng/mL**	5.95	5.1	0.8	.001	<.001
**Use of calcium supplements, no. (%)**	13 (12)	17 (11)	37 (16)	.66	.40
**Use of multivitamins, no. (%)**	43 (40)	62 (38)	101 (43)	.71	.65
**Intake, mean (SD)**					
Total calories, kcal/d	2,098 (1,197)	1,879 (876)	1,811 (819)	.40	.14
Total calcium, mg/d	797 (473)	797 (478)	825 (512)	.90	.73
Calcium from food, mg/d	690 (413)	706 (408)	692 (399)	.52	.79

Abbreviations: SD, standard deviation; BMI, body mass index; MET, metabolic equivalents; PSA, prostate-specific antigen.

a Indicates difference between cases and biopsy-negative controls; calculated by using χ^2^ test for categorical variables and Wilcoxon rank sum test for continuous variables.

b Indicates difference between cases and healthy controls; calculated by using χ^2^ test for categorical variables and Wilcoxon rank sum test for continuous variables.

**Table 2:** Calcium and Vitamin D Intakes and Supplement Use Among Controls, by Race, Among Veterans Screened for Prostate Cancer at Durham Veterans Affairs Medical Center, 2007-2010

Intake	Biopsy-Negative Controls			Healthy Controls		

	Blacks (n = 66)	Whites (n = 89)	*P* Value^a^	Blacks (n = 82)	Whites (n = 148)	*P* Value^a^
Total calcium, mean (SD), mg/d	677 (380)	873 (508)	.02	732 (452)	880 (540)	.06
Calcium from food, mean (SD), mg/d	619 (368)	759 (405)	.04	639 (420)	722 (383)	.04
Use calcium supplements, %	10	11	.82	16	18	.76
Use multivitamins, %	32	47	.06	47	44	.65
Total calories, mean (SD), kcal/d	1,821 (961)	1,908 (787)	.36	1,726 (931)	1,877 (757)	.07

Abbreviation: SD, standard deviation.

a Calculated by using χ^2^ test for categorical variables and Wilcoxon rank sum test for continuous variables.

**Table 3:** Dietary Calcium Intake and Risk for Prostate Cancer Among Veterans Screened for Prostate Cancer at Durham Veterans Affairs Medical Center, 2007-2010

Cases vs Biopsy-Negative Controls						

Median Intake	All Races^a^ (n = 269)		Black (n = 126)		White (n = 136)	

	No. of Cases	OR (95% CI)^b^	No. of Cases	OR (95% CI)^b^	No. of Cases	OR (95% CI)^b^
**Total calcium, mg/d**						
Tertile 1^c^: 376.8	48	1 [Reference]	37	1 [Reference]	11	1 [Reference]
Tertile 2: 704.7	28	0.85 (0.45-1.63)	10	0.43 (0.17-1.11)	17	1.73 (0.54-4.58)
Tertile 3: 1,174.8	32	0.85 (0.45-1.61)	13	0.53 (0.21-1.34)	19	1.70 (0.66-4.41)
*P* value for linear trend^d^	.66		.17		.37	
**Calcium from food, mg/d**						
Tertile 1^c^: 367.3	43	1 [Reference]	28	1 [Reference]	15	1 [Reference]
Tertile 2: 597.3	44	1.28 (0.70-2.37)	22	1.03 (0.43-2.43)	21	1.58 (0.65-3.86)
Tertile 3: 1,093.8	21	0.54 (0.27-1.05)	10	0.53 (0.20-1.43)	11	0.61 (0.23-1.60)
*P* value for linear trend^d^	.05		.22		.22	

**Cases vs Healthy Controls**						

**Median Intake**	**All Races^a^ (n = 345)**		**Black (n = 142)**		**White (n = 195)**	

	**No. of Cases**	**OR (95% CI)^b^**	**No. of Cases**	**OR (95% CI)^b^**	**No. of Cases**	**OR (95% CI)^b^**

**Total calcium, mg/d**						
Tertile 1^e^: 390.6	50	1 [Reference]	39	1 [Reference]	11	1 [Reference]
Tertile 2: 707.5	29	0.67 (0.37-1.21)	8	0.25 (0.10-0.67)	20	1.61 (0.69-3.78)
Tertile 3: 1,245.9	29	0.60 (0.33-1.08)	13	0.39 (0.16-0.95)	16	1.14 (0.47-2.76)
*P* value for linear trend^d^	.11		.04		.98	
**Calcium from food, mg/d**						
Tertile 1^e^: 346.4	54	1 [Reference]	37	1 [Reference]	17	1 [Reference]
Tertile 2: 602.2	31	0.72 (0.40-1.29)	13	0.48 (0.20-1.15)	17	0.92 (0.41-2.11)
Tertile 3: 1,054.5	23	0.50 (0.27-0.91)	10	0.42 (0.17-1.05)	13	0.63 (0.27-1.46)
*P* value for linear trend^d^	.02		.06		.27	

Abbreviations: OR, odds ratio; CI, confidence interval.

a Includes black, white, and other races (n = 7).

b Adjusted for age, total calories, race (in combined analyses), and prostate-specific antigen (in analyses of prostate cancer cases vs biopsy-negative controls).

c We created categories of calcium intake based on tertiles of intake among biopsy-negative controls.

d *P* values for linear trend were based on the median intake of each tertile, which was subsequently modeled as a continuous variable.

e We created categories of calcium intake based on tertiles of intake among healthy controls.

**Table 4:** Dietary Calcium Intake and Risk for Low-Grade and High-Grade Prostate Cancer Among Veterans Screened for Prostate Cancer at Durham Veterans Affairs Medical Center, 2007-2010

Median Intake	Low-Grade Prostate Cancer vs Biopsy-Negative Controls		High-Grade Prostate Cancer vs Biopsy-Negative Controls	

	No. of Cases	OR (95% CI)^a^	No. of Cases	OR (95% CI)^a^
**Total calcium, mg/d^b^**				
Tertile 1^c^: 376.8	21	1 [Reference]	27	1 [Reference]
Tertile 2: 704.7	19	1.39 (0.64-3.05)	9	0.41 (0.16-1.03)
Tertile 3: 1,174.8	20	1.27 (0.59-2.72)	12	0.46 (0.20-1.09)
*P* value for linear trend^d^	.62		.11	
**Calcium from food, mg/d**				
Tertile 1^c^: 367.3	18	1 [Reference]	25	1 [Reference]
Tertile 2: 597.3	30	2.25 (1.06-4.76)	14	0.60 (0.26-1.37)
Tertile 3: 1,093.8	12	0.74 (0.31-1.73)	9	0.37 (0.15-0.90)
*P* value for linear trend^d^	.33		.02	

**Median Intake**	**Low-Grade Prostate Cancer vs Healthy Controls**		**High-Grade Prostate Cancer vs Healthy Controls**	

	**No. of Cases**	**OR (95% CI)^a^**	**No. of Cases**	**OR (95% CI)^a^**

**Total calcium, mg/d^b^**				
Tertile 1^e^: 390.6	23	1 [Reference]	27	1 [Reference]
Tertile 2: 707.5	20	1.11 (0.54-2.28)	9	0.34 (0.14-0.80)
Tertile 3: 1,245.9	17	0.83 (0.40-1.73)	12	0.40 (0.18-0.90)
*P* value for linear trend^d^	.56		.04	
**Calcium from food, mg/d**				
Tertile 1^e^: 346.4	25	1 [Reference]	29	1 [Reference]
Tertile 2: 602.2	22	1.24 (0.61-2.52)	9	0.33 (0.14-0.79)
Tertile 3: 1,054.5	13	0.63 (0.29-1.36)	10	0.38 (0.17-0.86)
*P* value for linear trend^d^	.21		.02	

Abbreviations: OR, odds ratio; CI, confidence interval.

a Adjusted for age, total calories, race, and prostate-specific antigen (in analyses of prostate cancer cases vs biopsy-negative controls).

b Total dietary calcium intake includes calcium from food and from supplements.

c We created categories of calcium intake based on tertiles of intake among biopsy-negative controls.

d *P* values for linear trend were based on the median intake of each tertile, which was subsequently modeled as a continuous variable.

e We created categories of calcium intake based on tertiles of intake among healthy controls.
